# A non-lethal method for studying scorpion venom gland transcriptomes, with a review of potentially suitable taxa to which it can be applied

**DOI:** 10.1371/journal.pone.0258712

**Published:** 2021-11-18

**Authors:** Freek J. Vonk, Mátyás A. Bittenbinder, Harald M. I. Kerkkamp, Dwin G. B. Grashof, John P. Archer, Sandra Afonso, Michael K. Richardson, Jeroen Kool, Arie van der Meijden

**Affiliations:** 1 Naturalis Biodiversity Center, Leiden, The Netherlands; 2 Faculty of Sciences, Division of BioAnalytical Chemistry, Department of Chemistry and Pharmaceutical Sciences, Amsterdam Institute of Molecular and Life Sciences, Vrije Universiteit Amsterdam, Amsterdam, The Netherlands; 3 Animal Science and Health Cluster, Institute of Biology Leiden, Leiden University, Leiden, The Netherlands; 4 CIBIO-InBIO, Biopolis, Universidade do Porto, Porto, Portugal; Weizmann Institute of Science, ISRAEL

## Abstract

Scorpion venoms are mixtures of proteins, peptides and small molecular compounds with high specificity for ion channels and are therefore considered to be promising candidates in the venoms-to-drugs pipeline. Transcriptomes are important tools for studying the composition and expression of scorpion venom. Unfortunately, studying the venom gland transcriptome traditionally requires sacrificing the animal and therefore is always a single snapshot in time. This paper describes a new way of generating a scorpion venom gland transcriptome without sacrificing the animal, thereby allowing the study of the transcriptome at various time points within a single individual. By comparing these venom-derived transcriptomes to the traditional whole-telson transcriptomes we show that the relative expression levels of the major toxin classes are similar. We further performed a multi-day extraction using our proposed method to show the possibility of doing a multiple time point transcriptome analysis. This allows for the study of patterns of toxin gene activation over time a single individual, and allows assessment of the effects of diet, season and other factors that are known or likely to influence intraindividual venom composition. We discuss the gland characteristics that may allow this method to be successful in scorpions and provide a review of other venomous taxa to which this method may potentially be successfully applied.

## Introduction

Venoms are complex mixtures of bioactive compounds called toxins that have evolved on more than 30 different occasions in the animal kingdom [1,2]. Toxins are often highly specific in their activity and can induce a wide range of pharmacological effects [2–6]. They can act by binding to ion-channels for example, by destroying cellular components or by disrupting metabolic pathways, which may lead to paralysis, haematological disruptions, tissue necrosis and pain. Venoms have been studied for a long time in order to better understand their physiological effects from a standpoint of development of treatment of envenomation. However, the high specificity and potency of certain toxins renders them useful as experimental tools or as candidates for the development of novel therapeutics [1,7–12]. The field of transcriptomics has changed our understanding of the diversity and composition of animal venoms in the last decades. Venom transcriptome research focusses primarily on studying venom composition through mRNA-transcripts and their relative expression levels [11,13–16]. The main advantage of using transcriptomics over proteomics is the fact that the transcriptome data allows the study of patterns of gene activation through read counts, and gene evolution through the coding sequences.

Scorpions are, apart from snakes, the most widely studied group of venomous animals when it comes to their venom [4,14,17–20]. This is because scorpion stings are considered a public health problem in (sub-)tropical countries, with global estimates surpassing 1.2 million cases of envenoming resulting in more than 3,250 fatalities a year [5,17]. Scorpion venoms are also studied as candidates of pharmaceutically active molecules with potential drug applications [21–24]. Scorpion venoms are complex mixtures of proteins, peptides and small molecular compounds, with small peptides and proteins targeting ion channels being most prevalent [4,5]. Because of their high specificity for these ion channel proteins, scorpion toxins are being studied for their potential use as therapeutics. As these ion channel proteins are major drug targets, scorpion venoms could be an interesting source for novel candidates in the venoms-to-drugs pipeline [21–23]. Scorpion venoms have been studied via proteomic approaches within the last two decades, but with the emergence of next generation sequencing (NGS) technologies, transcriptome studies have increased in popularity [14,25–35]. The fact that the costs of next generation sequencing have decreased tremendously in recent years has revolutionized ‘omics’-studies [11,36–44]. The current method for obtaining a scorpion venom gland transcriptome is based on sacrificing the animal to extract the venom gland from the telson (the “stinger” at the end of the tail-like metasoma, containing the venom glands) or to homogenise the entire telson. Although this method of venom gland sequencing allows an in-depth analysis of the expressed genes in the venom gland, it has its limitations. One of the major limitations is the fact that this only allows analysis at a single time point, making it impossible to study the intraindividual variation caused by ontogenetic stage, season or diet [45,46]. Homogenisation of the telson also includes tissues not involved in venom production, such as the muscles surrounding the glands, and the cuticle, making the sequencing less target-specific. Furthermore, it faces the ethical drawbacks and resource depletion of having to sacrifice the animal [47].

In this study, we have performed mRNA-extraction from the scorpion venom itself, which can be done by using venom that is obtained by electro-stimulation without harming the animal, followed by standard sequencing and analysis techniques. This was done without amplification of mRNA-transcripts in order to avoid bias in the read counts. The novel technique allows for multiple time point transcriptomes from a single individual. This means that we can look at patterns of gene activation over time in the same individual, and assess the effects of diet, season and other factors that are known or likely to affect intraindividual venom composition [45,46]. This study compares venom-derived transcriptomes to the traditional whole-telson transcriptomes in order to show that they are similar. In addition, we will show an extraction at an earlier stage in the gland replenishment to show the possibility of doing a multiple time point extraction using this new method.

## Methods

### Tissue samples

The tissue and venom samples were obtained from a captive specimen of *Heterometrus laoticus* Couzijn, 1981, obtained from Vietnam through the pet trade. The specimen was maintained in the laboratory as described in [48]. Electrostimulation to obtain venom was performed by applying a square wave with an amplitude of 18V and a 10% duty factor at a frequency of 45Hz through saline-wetted electrodes positioned at the 2nd and 5th metasomal segments. We found this method to be scorpion-friendly, contrary to methods that result in high current through the tissues, such as those employing higher voltages or constant current (see S1 File for a schematic of the device and accompanying code).

To ensure active transcription of venom genes, the venom glands were first emptied by electrostimulation. This venom was discarded. Venom was extracted again 4 days after. The venom from this second extraction was frozen in liquid nitrogen and stored at -80°C until library preparation. The specimen was then fed and not disturbed for 2 weeks. Venom was then extracted again to stimulate transcription by emptying the venom glands, and this venom was discarded. The venom that was extracted 2 days later was frozen in liquid nitrogen and stored at -80°C until library preparation. After another rest period of 2 weeks, venom was extracted and discarded again. Five days after this extraction, the specimen was anaesthetised using isoflurane, and frozen in liquid nitrogen. The telson and chela were removed, and stored separately at -80°C until library preparation.

### cDNA library construction

The RNA extractions were done using the RNeasy Mini Kit (Qiagen) according to the manufacturer’s instructions. Then, RNA quantification was performed using the Qubit RNA BR (Broad-Range) Assay Kit (Thermo Fisher). The RNA quality was assessed by doing a RIN test using a Tapestation 2200 (Agilent). Library prep was done using the TruSeq RNA Library Prep Kit v2 (Illumina).

### DNA sequencing and bioinformatics analysis

The RNA samples were sequenced on an Illumina Hiseq 1500. All samples were given a unique index sequence with read lengths of ~ 280 bp. Sequences were pair-ended (2 × 125 bp). The venom samples, chela and telson were sequenced separately resulting in four transcriptomes in total. To annotate the telson transcriptomes a custom pipeline was constructed, largely in BioPython (version 1.70), a module for Python (version 3.6.4). This custom bioinformatics pipeline follows six steps leading to full annotation of the transcriptomes: (i) The pipeline calculates the coverage by: average read length (150) * read count of the transcript / length of the transcript. With this formula the coverage of a single transcript is normalised by its size, making the transcript coverage comparable to each other. (ii) The pipeline removes orthologues between the telson and chela transcriptomes by performing a BLASTn with the following parameters: e-value = 1e¬-50; output format = 6; max subject sequences = 1; minimal percentage identity = 99%; minimal percentage coverage = 95%. This removes most housekeeping transcripts from the telson transcriptome. Since no venom or toxin genes are likely to be expressed in the chela, all toxin transcripts, together with some physiological transcripts not expressed in the chela, are kept in the telson transcriptome. (iii) For every transcript left in the transcriptome an open reading frame (ORF) is predicted to increase speed, accuracy and relevance of the next steps. (iv) BLASTp annotation. The fourth step is the actual annotation using BLASTp and the previously created annotated database filled with both physiological and toxin arthropod proteins. The ORF of every transcript is blasted against the annotated database, using the parameters: e-value 1e-5; output format = 6; max subject sequences = 1. The transcripts are then labelled based on the label of their BLASTp hit or considered “unidentified”. (v) Transcript ORF have to be uploaded to SignalP to predict their signal peptides with the SignalP sensitivity set on “Sensitive” [49]. Since this requires manual input, this step was interchanged with step vi to reduce the amount of manual work. (vi) The last step of this pipeline uses all previously gathered data to label the transcripts. Transcripts with a coverage value lower than 5 were removed, since those transcripts have a higher chance of being misassembled and are assumed to be insignificant in the venom of the scorpion. Then all transcripts that were found to have an orthologue in the chela transcriptome were labelled as “physiological”. Finally, the remaining transcripts were labelled according to the label of their BLASTp hit. The transcripts labelled as a member of a toxin family or labelled as “other toxin” were considered part of the venom.

## Results

**Fig 1:**
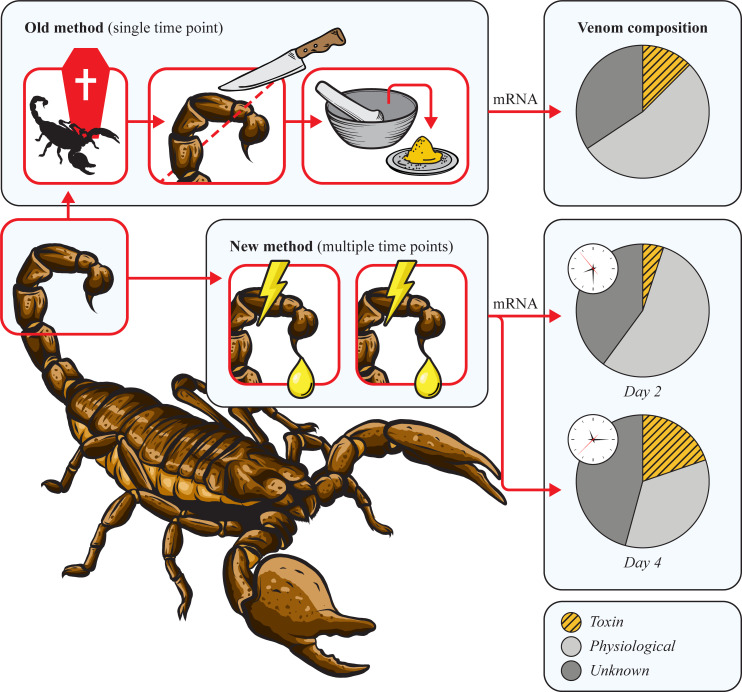
Schematic overview of the old method and the here presented new method for the generation of venom gland transcriptomes. Comparison between the ‘traditional’ method and our newly developed method for the extraction of mRNA from the Vietnam forest scorpion (*Heterometrus laoticus*). The traditional method involves sacrificing the animal to extract the venom gland from the telson or to homogenize the entire telson in order to collect mRNA, whereas the newly developed method includes mRNA-extraction from scorpion venom, which overcomes the need of harming the animal.

**Fig 2:**
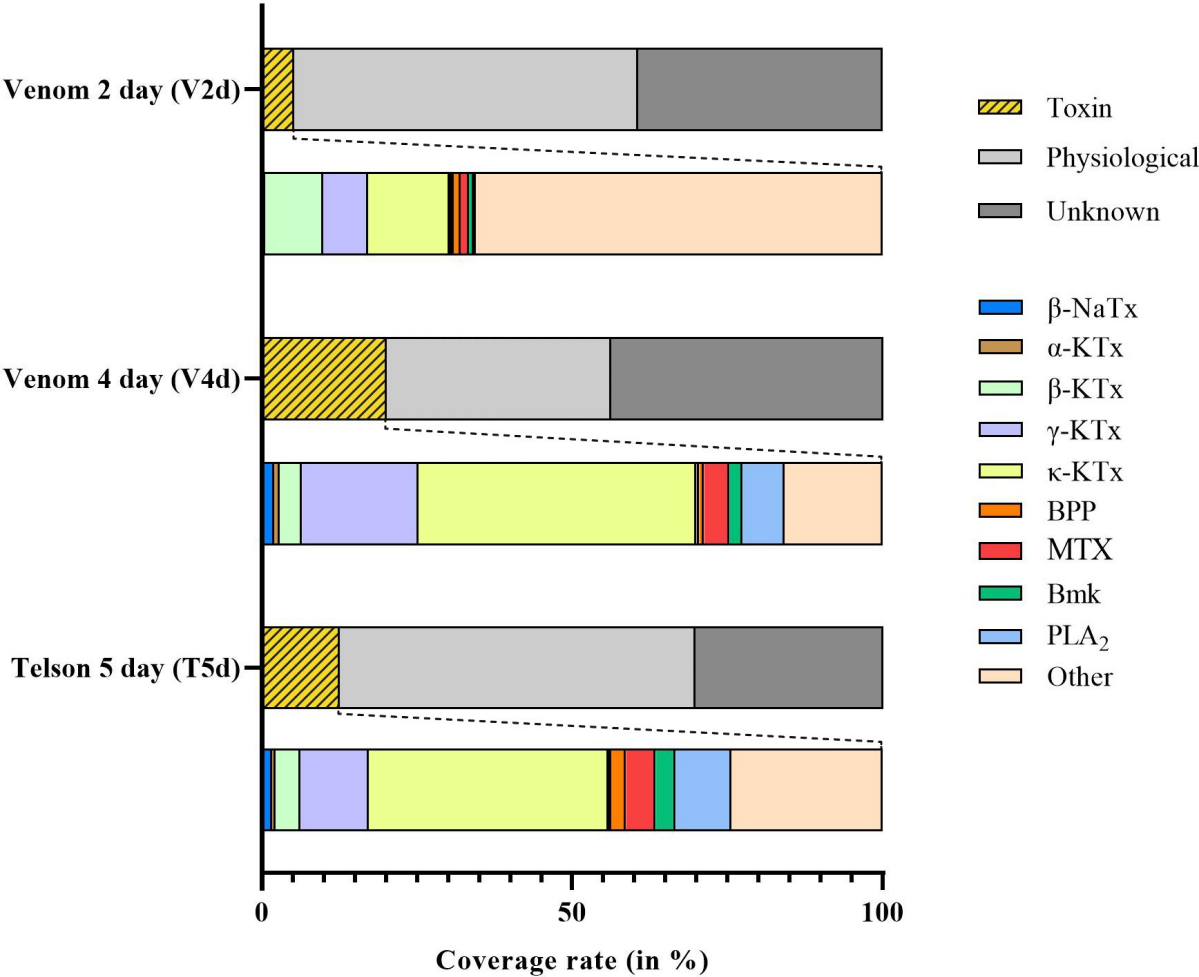
Relative expression levels of transcripts in the venom gland transcriptomes at different timepoints. The graphs show expression rates for venom gland transcriptomes two days after start of venom replenishment (V2d) and four days after the start of venom replenishment (V4d) relative to the whole telson transcriptome five days after the start of venom replenishment (T5d). In the upper bar graphs the transcripts are labelled as “toxin”, “physiological” and “unknown” and are shown as a percentage of total expression. The lower graphs represent the relative expression levels across toxin families within the “toxin” transcripts in the venom gland transcriptomes. Abbreviations: NaTx (sodium-channel binding toxin); KTx (potassium-channel binding toxins); ClTx (chlorotoxin); BPP (bradykinin-potentiating peptide); MTX (Maurotoxin); BmK (*Buthus martensii* Karsch-toxin); PLA_2_ (phospholipase A_2_). Note: Some toxins are found at such low expression rates that these differentiated from the bar graphs, therefore these are not represented in the legend. These include α-NaTx, calcium-channel binding toxin and Kunitz-type toxin. Host defense proteins were classified under “other”.

In this study we introduce a new method for the generation of venom gland transcriptomes by using extracted scorpion venom from the Vietnam forest scorpion (*Heterometrus laoticus*) (Fig 1). We extracted venom at two days after the start of venom replenishment (V2d) and at four days after the start of venom replenishment (V4d). From these two samples we extracted the mRNA and using Illumina sequencing we generated two venom transcriptomes (Fig 1). Next, using the old method by extracting mRNA from tissue, we generated a telson transcriptome of five days after the start of venom replenishment (T5d) (Fig 2). These venom gland transcriptomes were BLASTed and processed using our python pipeline [35]. Our extractions of the venom gland, the chela, V2d and V4d resulted in 1.32, 0.208, 0.30, 0.678 μg of RNA respectively. However, since we did not standardize the tissue/venom volume used in the extractions, these quantities do not correspond to the RNA quantity in the original tissue or venom.

When we label genes belonging to a toxin family as “toxin”, other genes as “physiological” and the remaining unidentified transcript as “unknown”, we find that of the transcripts in the V2d transcriptome 5% are labelled “toxin”, 55% are labelled “physiological” and 40% are considered “unknown”, whereas in the V4d transcriptome 20% are labelled “toxins”, 36% are labelled “physiological” and 44% are considered “unknown”. Lastly, in the T5d transcriptome 13% of the transcript are labelled as “toxins”, 58% are labelled “physiological” and 30% are considered “unknown” (Fig 2). When comparing the individual toxin families present in the venom transcripts, we find that the majority consists of “other toxins” (i.e., venom components not belonging to a major toxin family), followed by potassium-channel binding toxins (KTxs), and phospholipase A_2_s (PLA_2_s) (Fig 2). The V2d transcriptome, is mostly made up of “other toxins” (65.5%), followed by κ-KTxs (13.1%), β-KTxs (9.3%) and γ-KTxs (7.2%) (Fig 2). The majority of the V4d transcriptome consists of κ-KTxs (44.7%), followed by γ-KTxs (18.8%), “other toxins” (15.8%) and PLA_2_s (6.7%) (Fig 2). Lastly, the T5d transcriptome is largely comprised of κ-KTxs (38.6%), followed by “other toxins” (24.4%), γ-KTxs (11.0%) and PLA_2_s (8.9%) (Fig 2). The total number of read counts for each transcriptome analysis (sum read per contig) consisted of: 42,616,910 reads (V2d), V4d: 37,077,624 reads (V4d), 48,491,348 reads (T5d).

## Discussion

This paper describes a new way of generating a scorpion venom gland transcriptome, without sacrificing the animal, using RNA found in extracted venom. This permits the study of the transcriptome at various time points within a single individual. We have used our method to generate two venom gland transcriptomes suitable for toxin analysis. In order to make a comparison between the two methods, we looked at the V4d and T5d results as these are most similar in point of venom replenishment. Ideally, it would have been more accurate to compare the venom gland transcriptome data (T5d) with the venom transcriptome at the same number of days after venom extraction. However, at day four and five, the venom replenishment is operating fully, as this is in the middle of the duration reported for venom gland replenishment. At this point, the gland is not yet filled, and production of peptides should be maximal. Since it takes time to start the production of the many peptides and glycoproteins that make up scorpion venom, we expect the largest differences in gene activation to lie in the first days of venom replenishment. This is why we expected the difference between day two and four to be larger than between day four and five, as indeed it seems to be. Nevertheless, our results show that this method shows differences in gene activation at different time points after gland depletion, and that these venom transcriptomes are equally informative as the traditional gland transcriptomes.

When comparing their relative toxin composition, we find that the V4d venom transcriptome mostly consists of κ-KTxs, γ-KTxs, “other toxins” and PLA_2_s whereas the T5d transcriptome is made up primarily by κ-KTxs, “other toxins”, γ-KTxs and PLA_2_s (Fig 2). To further compare this, we aligned the protein sequences of κ-KTx and γ-KTx families (S1 and S2 Figs). Here we find that for the 33 κ-KTx sequences found in T5d, only three have no corresponding V4d sequence (S1 Fig). Furthermore, when looking at the expression of the individual sequences, we find that for sequences with high expression rate in the T5d transcriptome that their V4d counterpart also tends to have a higher expression rate (S1 and S2 Figs). This suggests that both methods result in similar expression rates in terms of toxin family, toxin sequences and individual sequence expression. However, there are some differences. These are possibly due to different time of extraction (and thus the difference in venom replenishment) or it is because of the method itself that these variations between transcriptomes occur.

Another concern with the proposed method is that as the mRNA is exposed to the extracellular environment of the venom gland, there is the possibility of RNA-degeneration. In order to examine the extent of RNA degeneration caused by the venom we did a contig comparison between V4d and T5d toxin transcripts. We aligned toxin transcripts with high expression of the major venom families and looked at the percentage of similarity for the overlapping parts. We found that although there are some differences, they are minor, as the similarity percentage is between 96% and 100% (S1 Table). This suggests there may be some RNA degeneration and although it is minimal, it is something that should be taken into account when considering using this method.

The generation of two venom transcriptomes of a single individual enabled us to compare ‘early’ venom replenishment to ‘late’ venom replenishment. V2d has a much lower relative amount of toxin transcripts compared to V4d, which may be explained by the fact that venom production had just started. When looking at the individual toxin families (Fig 2) we see that “other toxins” form the main component in the V2d transcriptome, representing 57% of toxin production. This is followed by κ-KTx (18%) and γ-KTx (11%), which are the main components (50% and 18% respectfully) in the V4d. This suggests that at two days the venom production is still getting started. Note that since expression is not standardized against housekeeping genes, these numbers should be interpreted with caution. The proposed method enables new studies into not just the gene activation at different time points of venom replenishment, but also the possible effects of diet or season on the venom production using repeated measurements in a single individual. Thereby this method allows the study of intraindividual variation and further overcomes the ethical drawbacks of having to sacrifice the animal.

**Fig 3:**
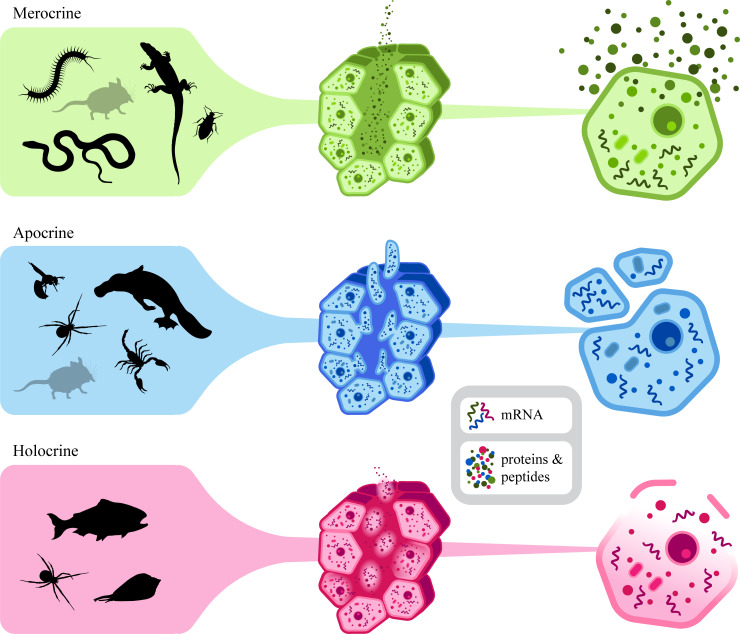
Schematic overview of the three secretion methods in the venom glands across the animal kingdom. The left panel shows the various venomous lineages, clustered based on the method of secretion of their venom-producing cells. For some lineages the gland type depends on the species and therefore these are represented multiple times in this figure. Note that some clades are shown in grey, as literature does not provide sufficient information to properly categorize these based on their method of venom secretion. The middle column of this figure shows a cartoon of excretory cells surrounding the gland lumen and right part of the figure schematically illustrates how venomous secretions are being produced by a single cell (see text for further details).

This method is based on the fact that mRNA-transcripts for the toxins present in the venom can be used to perform transcriptomic analysis. Venom gland histology plays an important role in the possibility of performing transcriptomics on the mRNA-transcripts present in the venom. Gland products are secreted by exocrine glands onto an epithelial surface, as opposed to products of endocrine glands that are released into the blood stream [50–54]. Three types of exocrine venom glands are recognised, depending on how the venom is being secreted (Fig 3). The first type are the merocrine glands. With this type of secretion, the venom is released through exocytosis without part of the gland cells being lost or damaged. However, cytoplasm and cellular debris can still enter the gland lumen, although only when cells rupture or die [50,54]. These gland types are found in centipedes, heteropterans, reptiles and some mammals (Fig 3, Table 1) [51,55–58]. The second type of glands have an apocrine secretion, in which parts of the cell bud off, producing membrane-bound vesicles containing the cytoplasm of the cell [50,54]. This cytoplasm contains both the venom components as well as cytoplasm containing various cell-specific components such as mRNA-transcripts. This secretion type is found in scorpions, spiders, hymenopterans (i.e. wasps, bees and ants), and the enigmatic platypus (Fig 3, Table 1) [13,59–66]. The third secretion type, in which the accumulation of secretion (i.e., venom components) in the cytoplasm of the secretory cells cause disintegration of the entire cell, is referred to as holocrine secretion. The venom secretions which have accumulated in the cell are released into the gland lumen by rupture of the cell membrane. This type of secretion also delivers cytoplasm, cellular organelles and nuclear cell products into the lumen of the gland, including mRNA [50,51,54,67]. These types of glands can be found in the venom glands of cone snails, certain spider species and teleost fish (Fig 3, Table 1) [59,60,68–77]. Spiders utilise both apocrine and holocrine secretion mechanisms, depending on the species [13,60,61,78]. The secretion method in the venom glands of mammals is dependent on the clade to which these mammals belong. Venomous insectivores (i.e. solenodons and shrews) have evolved an oral venom system that closely resembles the submaxillary salivary glands, suggesting a merocrine secretion type [44,79]. In male platypuses on the contrary, venom is produced in glands on an extratarsal spur on each hind leg. These glands are likely to have been derived from modified apocrine sweat glands [66,80]. Lastly, the venom secretion of slow and pygmy lorises is rather unique, as these venoms comprise a combination of saliva (merocrine secretion) and fluid from the brachial gland (apocrine secretion) [81,82].

**Table 1:** Overview of different methods of venom gland secretion across all venomous lineages.

Type of gland	Cytoplasm released?	Animal groups	References
Merocrine	No	Centipedes	[57,83–85]
		Heteropterans (assassin bugs, giant water bugs)	[51,58,86]
		Reptiles (snakes, lizards)	[55,56,74]
		Mammals (insectivores)	[44,79]
Apocrine	Yes	Scorpions	[59,87–89]
		Spiders	[13,60,61,78]
		Hymenopterans (bees, ants, wasps)	[62–64]
		Mammals (platypus)	[66,80]
Holocrine	Yes	Cone snails	[68,69,73]
		Spiders	[13,60,61,78]
		Teleost fish	[70–72,74,90,91]
Miscellaneous		Cephalopods*	[43,65,92]
		Cartilaginous fish (stingrays, venomous sharks, chimaeras)*	[67,93–95]
		Mammals (slow lorises, pygmy lorises)**	[81,82]

The various venomous lineages are categorized based on the secretion mechanism in the venom gland. Note that for some taxa the gland type depends on the species and therefore these are represented multiple times in this table. For some clades literature does not provide sufficient information to properly categorize them based on their method of secretion. These have been listed under “miscellaneous”.

*No convincing distinction can be made on present literature

**The venom of lorises is a combination of merocrine and apocrine secretions.

For some venomous clades, literature is lacking or in some cases even contradictory when it comes to classifying these groups based on their method venom secretion. Therefore, no convincing distinction can be made based on present literature. This is the case for the venom secretion mechanisms of cephalopods and cartilaginous fish. Consequently, categorising these clades based on secretion mechanisms should be done with caution, as some gland types are considered apocrine in certain papers, whereas other papers classify these as holocrine. In some cases, the presence of degenerating secretory cells might have been erroneously interpreted as proof for holocrine secretion, whereas the actual mode of secretion is apocrine or even merocrine. This may be the case in the glands of scorpions and cartilaginous fish [59,74,77,87,89]. The same misinterpretation was probably also present in some studies of the ultrastructure of centipede venom glands, in which merocrine secretory glands were wrongfully interpreted as being holocrine [57,83–85].

Animals carrying venom glands with merocrine cells deliver the venom components through exocytosis instead of releasing cytoplasm into the lumen [55,56]. This might explain why mRNA-yields from snake venom are low [96,97]. The excretion type found in these animals makes it impossible to perform NGS of the venom gland without amplification of the mRNA-transcripts. Chen *et al*. managed to sequence mRNA from amphibian skin and snake venom using rtPCR [98,99]. These studies were followed up by studies that were using NGS methods to amplify mRNA in snake venom [96,97]. These studies concluded that snake venom does not contain sufficient transcripts for NGS sequencing without amplification. Such amplification could introduce unwanted bias in the read counts, making this method less feasible to quantify relative expression rates. Apocrine and holocrine secretion mechanisms on the contrary will potentially yield sufficient mRNA from the cytoplasm in the venom, which would make them suitable for transcriptomic analysis without amplification. In theory, all venoms that are being produced in apocrine or holocrine glands are potentially suitable for transcriptomic analysis. These excretory types will potentially yield mRNA from the cytoplasm in the venom. This paves the way for looking into the variety in venom composition, both intra-specifically and within the same individual. For scorpion venoms for example, we know that the composition exhibits a level of plasticity that can be influenced in response to environmental and behavioural factors [45,46]. Further, the process of venom replenishment is assumed to be an asynchronous process, with certain components being produced at different stages and at varying rates. With this new method, patterns of gene activation and venom production can be followed over time within the same individual, allowing us to study seasonal, ontogenetic and stress-related variation. Although this study focused on venom gland secretions primarily, poisonous animal lineages with apocrine or holocrine secretion methods (e.g., amphibians) might be similarly harnessed to generate the transcriptome of genome-derived compounds (i.e. proteins and peptides) in the poison glands [74–77,100].

## Supporting information

S1 Fig Alignment of κ-KTx contigs with their expression percentage. Overview of the alignments of the κ-KTx contigs from: Whole telson transcriptomes five days after start of venom replenishment (Heterometrus_VG); venom gland transcriptomes two days after start of venom replenishment (Heterometrus_2Day); venom gland transcriptomes four days after the start of venom replenishment (Heterometrus_4Day).
(DOCX)

S2 Fig Alignment of γ-KTx contigs with their expression percentage. Overview of the alignments of the γ-KTx contigs from: Whole telson transcriptomes five days after start of venom replenishment (Heterometrus_VG); venom gland transcriptomes two days after start of venom replenishment (Heterometrus_2Day); venom gland transcriptomes four days after the start of venom replenishment (Heterometrus_4Day).
(DOCX)

S1 Table Percentage of basepair similarity in overlapping parts of indicated T5d and V4d contigs. (DOCX)

S1 File A. Schematic overview of the scorpion venom extraction device. Schematic of the electrostimulator used to extract venom. This schematic includes an indicator LED, an on-off switch and a switch to choose between an amplitude of 9V and 18V, which all may be omitted for simplicity. The potentiometer is used to select frequency. The parallel 47Ω resistors limit the current to the scorpion. A switch may be added to either lead to the scorpion for fine control of the stimulus. Terminals at the metasoma should be wetted with a drop of saline solution to improve contact. B. Code for the Arduino microcontroller.
(DOCX)
