# Supplementary figures and images for: A non-lethal method for studying scorpion venom gland transcriptomes, with a review of potentially suitable taxa to which it can be applied
Source: PLoS One. 2021 Nov 18;16(11):e0258712. doi: 10.1371/journal.pone.0258712 (PMC8601437; doi:10.1371/journal.pone.0258712)

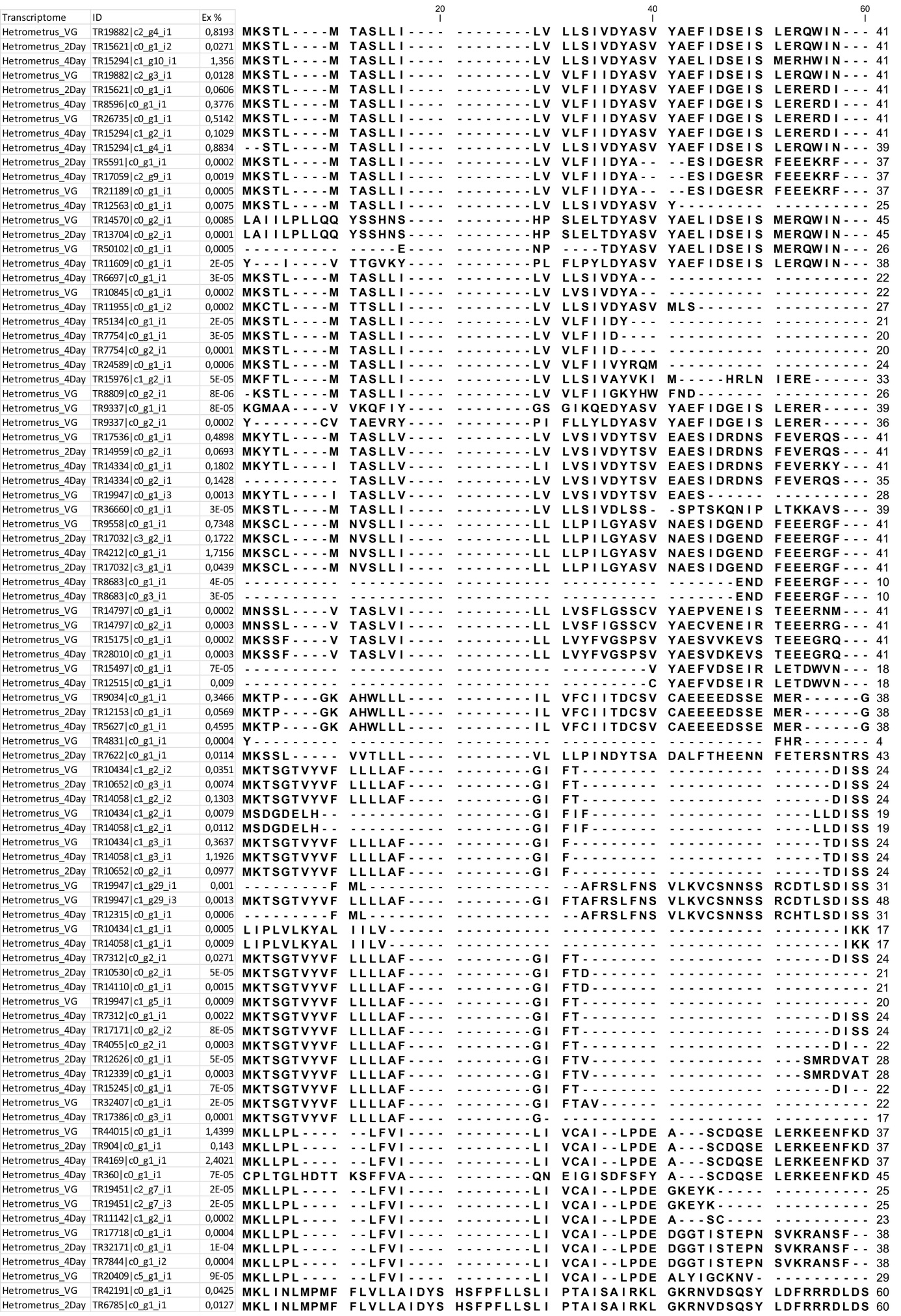


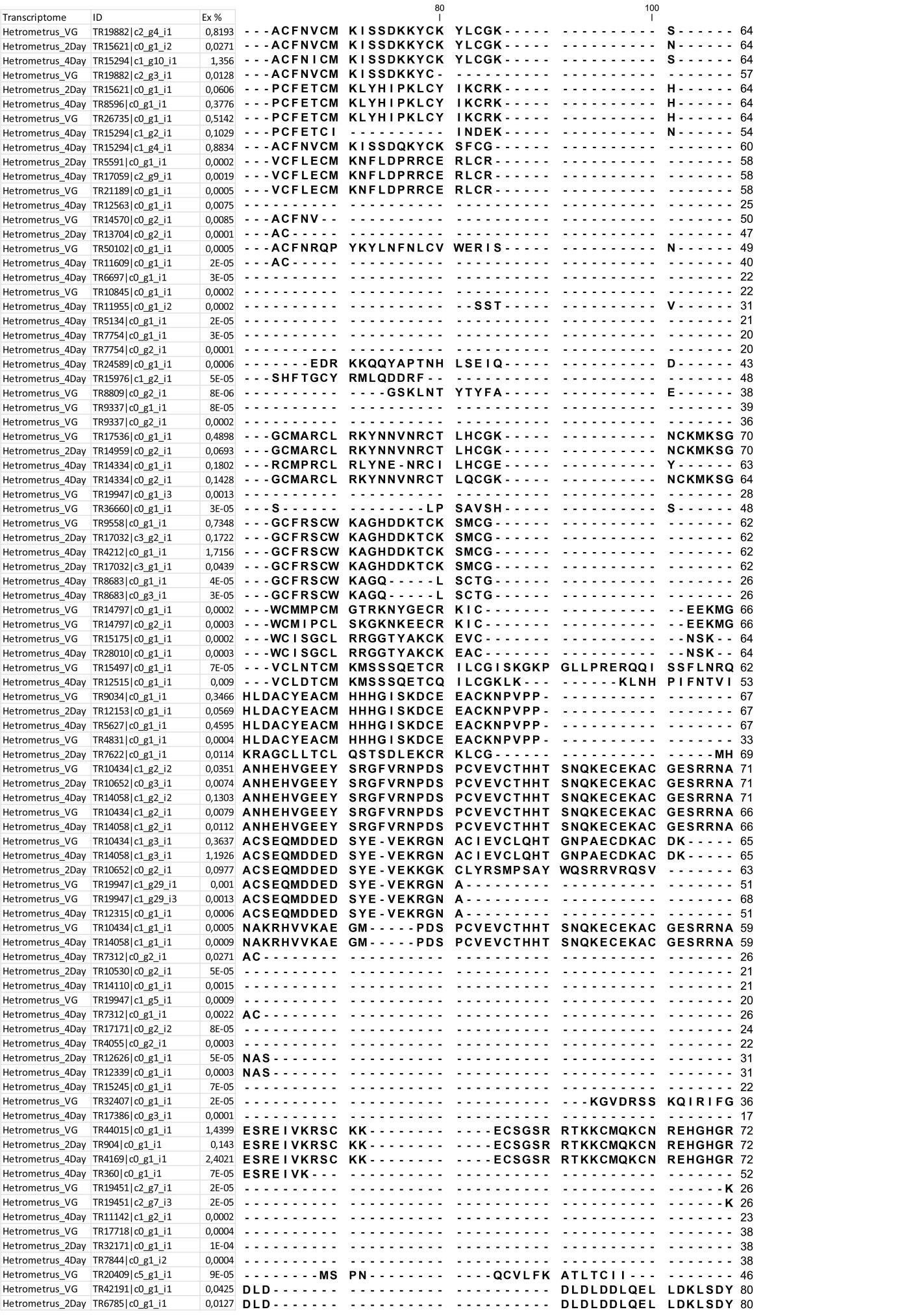

Supplement: S1 Fig — Overview of the alignments of the κ-KTx contigs from: Whole telson transcriptomes five days after start of venom replenishment (Heterometrus_VG); venom gland transcriptomes two days after start of venom replenishment (Heterometrus_2Day); venom gland transcriptomes four days after the start of venom replenishment (Heterometrus_4Day). (DOCX) [file pone.0258712.s001.docx]

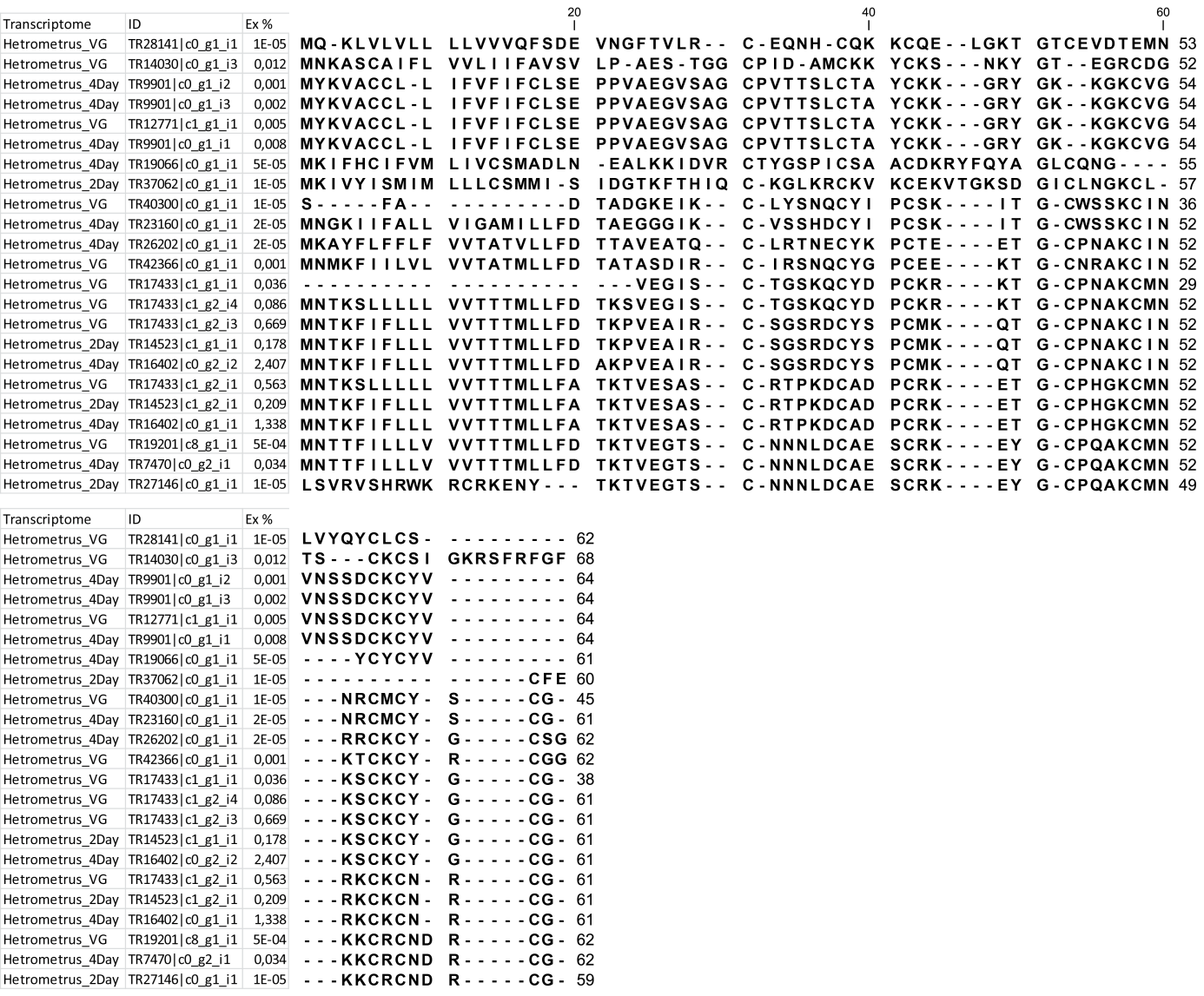

Supplement: S2 Fig — Overview of the alignments of the γ-KTx contigs from: Whole telson transcriptomes five days after start of venom replenishment (Heterometrus_VG); venom gland transcriptomes two days after start of venom replenishment (Heterometrus_2Day); venom gland transcriptomes four days after the start of venom replenishment (Heterometrus_4Day). (DOCX) [file pone.0258712.s002.docx]
