# Supplementary material for: A non-lethal method for studying scorpion venom gland transcriptomes, with a review of potentially suitable taxa to which it can be applied
Source: PLoS One. 2021 Nov 18;16(11):e0258712. doi: 10.1371/journal.pone.0258712 (PMC8601437; doi:10.1371/journal.pone.0258712)
Supplement: S1 Table — (DOCX) [file pone.0258712.s003.docx]

| **Toxin family** | **ID Td5 contig** | **ID V4d contig** | **Percentage similarity** |
| --- | --- | --- | --- |
| κ-KTx | TR44015\|c0_g1_i1 | TR4169\|c0_g1_i1 | 100 |
| κ-KTx | TR19882\|c2_g4_i1 | TR15294\|c1_g10_i1 | 94 |
| κ-KTx | TR9558\|c0_g1_i1 | TR4212\|c0_g1_i1 | 98 |
| γ-KTx | TR17433\|c1_g2_i3 | TR16402\|c0_g2_i2 | 97 |
| γ-KTx | TR17433\|c1_g2_i1 | TR16402\|c0_g1_i1 | 97 |
| γ-KTx | TR12771\|c1_g1_i1 | TR9901\|c0_g1_i1 | 100 |
| PLA_2_ | TR19508\|c0_g2_i4 | TR17239\|c3_g19_i1 | 96 |
| PLA_2_ | TR36880\|c0_g1_i1 | TR17239\|c3_g13_i1 | 95 |
| PLA_2_ | TR26410\|c0_g1_i1 | TR12676\|c0_g1_i1 | 100 |
