# Supplementary material for: A non-lethal method for studying scorpion venom gland transcriptomes, with a review of potentially suitable taxa to which it can be applied
Source: PLoS One. 2021 Nov 18;16(11):e0258712. doi: 10.1371/journal.pone.0258712 (PMC8601437; doi:10.1371/journal.pone.0258712)
Supplement: S1 File — A. Schematic overview of the scorpion venom extraction device. Schematic of the electrostimulator used to extract venom. This schematic includes an indicator LED, an on-off switch and a switch to choose between an amplitude of 9V and 18V, which all may be omitted for simplicity. The potentiometer is used to select frequency. The parallel 47Ω resistors limit the current to the scorpion. A switch may be added to either lead to the scorpion for fine control of the stimulus. Terminals at the metasoma should be wetted with a drop of saline solution to improve contact. B. Code for the Arduino microcontroller. (DOCX) [file pone.0258712.s004.docx]

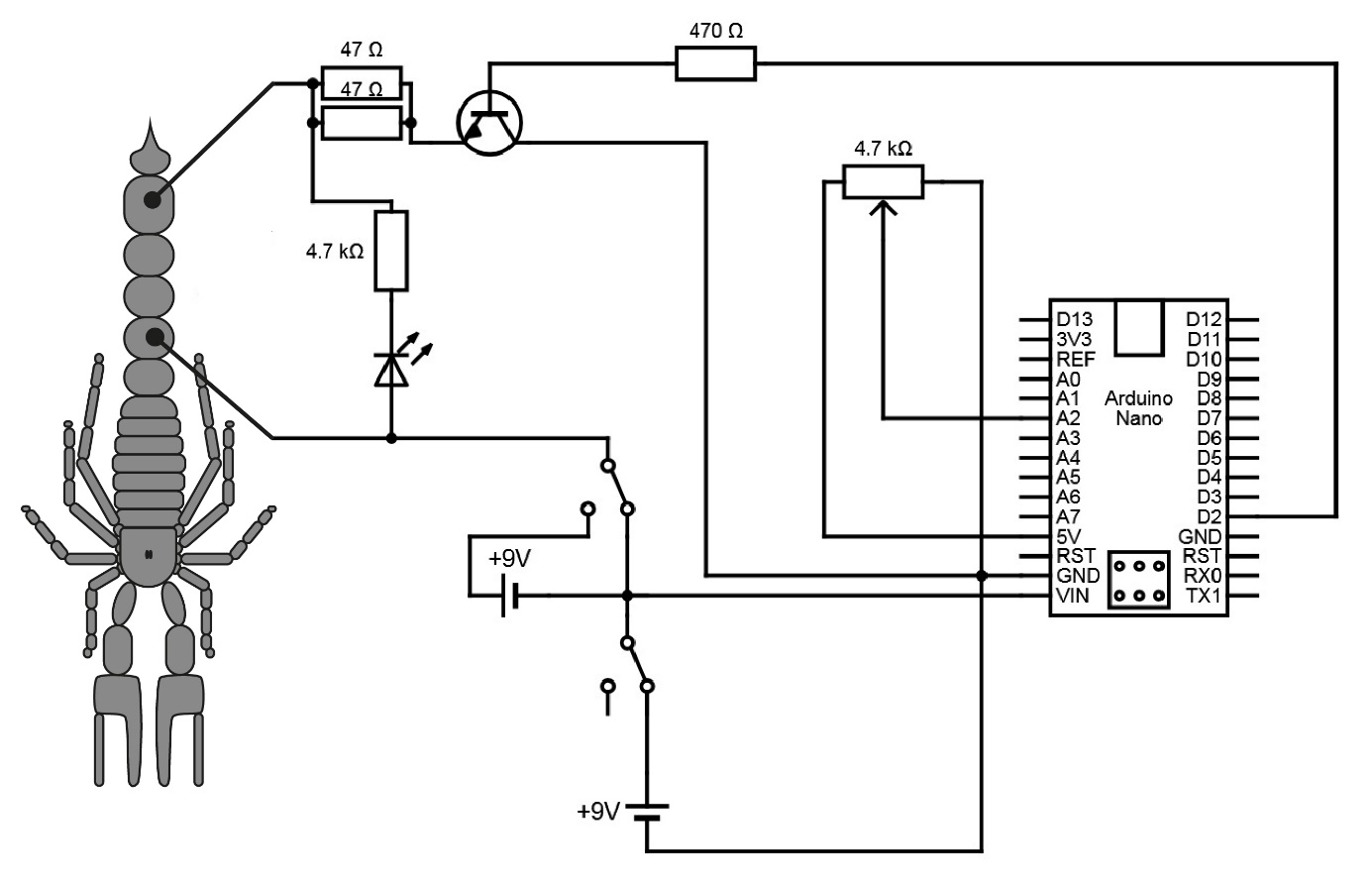


1. **Schematic overview of the scorpion venom extraction device.**
2. **Code for the Arduino microcontroller.**

// If you have any trouble, please contact [mail@arievandermeijden.nl](mailto:mail@arievandermeijden.nl)

int output = 2; // pin that controls the transistor

int freqpot = A2; // pin attached to potentiometer for frequency control

int freq = 40; // initial frequency in Hz

int df = 10; // duty factor in percent

int outputState = LOW; // set the output to low (no voltage)

long previousMillis = 0; // will store last time output was updated

int oninterval = 3; // duty factor in ms

int offinterval = 22;

// the setup routine runs once during startup or when you press reset:

void setup() {

pinMode(output, OUTPUT); // initialize the digital pin as an output.

pinMode(freqpot, INPUT); // initialize the analog pin as an input.

checkpot(); // potentiometer can only be changed at startup

}

//Function to check potentiometer for position to adjust frequency

void checkpot(){

int sensorValue = analogRead(freqpot);

freq = map(sensorValue, 0, 255, 10, 80); //You can change the frequency range here (currently 10-80Hz)

oninterval = (df/100)*(1/freq)*1000;

offinterval = ((1/freq)*1000)-oninterval;

}

void loop() {

//checkpot(); // Uncomment to check pots every cycle for adjustment of parameters. Incurs some time delay in the cycle

unsigned long currentMillis = millis();

switch (outputState) {

case LOW:

if(currentMillis - previousMillis > offinterval) {

// save the last time you switched the output

previousMillis = currentMillis;

outputState = HIGH;

}

break;

case HIGH:

if(currentMillis - previousMillis > oninterval) {

// save the last time you switched the output

previousMillis = currentMillis;

outputState = LOW;

}

break;

}

// set the LED with the ledState of the variable:

digitalWrite(output, outputState);

}
